# Improving Determination of Pigment Contents in Microalgae Suspension with Absorption Spectroscopy: Light Scattering Effect and Bouguer–Lambert–Beer Law

**DOI:** 10.3390/md21120619

**Published:** 2023-11-29

**Authors:** Yen-Cheng Yeh, Tobias Ebbing, Konstantin Frick, Ulrike Schmid-Staiger, Bernard Haasdonk, Günter E. M. Tovar

**Affiliations:** 1 Fraunhofer Institute for Interfacial Engineering and Biotechnology IGB, Nobelstraße 12, 70569 Stuttgart, Germany; tobias.ebbing@igvp.uni-stuttgart.de (T.E.); ulrike.schmid-staiger@igb.fraunhofer.de (U.S.-S.); guenter.tovar@igvp.uni-stuttgart.de (G.E.M.T.); 2 Institute of Interfacial Process Engineering and Plasma Technology, University of Stuttgart, Nobelstraße 12, 70569 Stuttgart, Germany; 3 Institute of Applied Analysis and Numerical Simulation, University of Stuttgart, Pfaffenwaldring 57, 70569 Stuttgart, Germany; haasdonk@mathematik.uni-stuttgart.de

**Keywords:** Bouguer–Lambert–Beer law, absorption spectroscopy, microalgae, light scattering, *Phaeodactylum tricornutum*, chlorophyll *a*, fucoxanthin, pigment content

## Abstract

The Bouguer–Lambert–Beer (BLB) law serves as the fundamental basis for the spectrophotometric determination of pigment content in microalgae. Although it has been observed that the applicability of the BLB law is compromised by the light scattering effect in microalgae suspensions, in-depth research concerning the relationship between the light scattering effect and the accuracy of spectrophotometric pigment determination remains scarce. We hypothesized that (1) the precision of spectrophotometric pigment content determination using the BLB law would diminish with increasing nonlinearity of absorbance, and (2) employing the modified version of the BLB (mBLB) law would yield superior performance. To assess our hypotheses, we cultivated *Phaeodactylum tricornutum* under varying illumination conditions and nitrogen supplies in controlled indoor experiments, resulting in suspensions with diverse pigment contents. Subsequently, *P. tricornutum* samples were diluted into subsamples, and spectral measurements were conducted using different combinations of biomass concentrations and path lengths. This was carried out to assess the applicability of the BLB law and the nonlinearity of absorbance. The chlorophyll *a* and fucoxanthin contents in the samples were analyzed via high-performance liquid chromatography (HPLC) and subsequently used in our modeling. Our findings confirm our hypotheses, showing that the modified BLB law outperforms the original BLB law in terms of the normalized root mean square error (NRMSE): 6.3% for chlorophyll *a* and 5.8% for fucoxanthin, compared to 8.5% and 7.9%, respectively.

## 1. Introduction

Photosynthesis is the process through which electromagnetic light energy is transformed into chemically bound energy in the form of various organic compounds within phototropic cells and organisms. In microalgal cells, pigments are specific chemical structures that are key components of this vital process. Chlorophylls play a pivotal role as the primary light-harvesting pigment in the photosynthesis of green algae and plants [1]. Chlorophyll *a* (Chl *a*) is tightly bound to antenna proteins within the thylakoid membrane of chloroplasts and is supported by chlorophyll *b*. In addition to chlorophylls, microalgae may contain a variety of other pigments, such as carotenoids and phycobilins. These pigments expand the action spectrum of photosynthesis by broadening the range of absorbable light wavelengths [2]. Furthermore, carotenoids play a significant role in light absorption and stress tolerance. For instance, the carotenoid fucoxanthin (Fx) in *Phaeodactylum tricornutum* is a pigment associated with photosynthesis, absorbing light in the blue–green spectrum. It acts as a light-harvesting pigment as it transfers the energy to the chlorophylls. In conditions of excess light, closely related carotenoids, diadinoxanthin and diatoxanthin, facilitate a process known as nonphotochemical quenching. This process dissipates excess energy generated during light harvesting, thereby safeguarding the cell from over-excitation [3]. Consequently, the precise measurement and monitoring of pigments is crucial to understanding changes in the pigmentation of microalgae, contributing to process optimization and improving light utilization in microalgae biotechnology.

In the current study, our primary focus lies on the spectrophotometric determination of chlorophyll *a* (Chl *a*) and fucoxanthin (Fx) contents within the marine diatom *P. tricornutum*. These pigments are bonded with the so-called fucoxanthin chlorophyll *a*/*c* binding protein (FCP) in *P. tricornutum* [4]. They display increased interest as an interesting bio-active product from microalgae and macroalgae. Chl *a* has various commercial applications in cosmetics and pharmaceutics [5]. Fx exhibits numerous health-beneficial traits [6], for example, in connection with nonalcoholic fatty liver disease [7,8]. Moreover, Fx shows anticancer, antioxidant, anti-inflammatory, and anti-obesity properties [9,10,11]. Fx from different sources is already being sold at high prices in nutraceuticals. Furthermore, the interest in producing carotenoids is still of growing interest, especially in microalgae biotechnology [12]. Notably, research has demonstrated that Fx, as a high-value product, plays a pivotal role in rendering microalgae production of *P. tricornutum* economically viable [13].

Conventional methods for quantifying pigment include a series of laboratory methods such as lyophilization, cell disruption, extraction, and measurement using high-performance liquid chromatography (HPLC), which can take from hours to days for the whole process [14,15]. Several interesting optical methods have been developed for rapid pigment estimation using absorption/transmission spectroscopy, Raman spectroscopy, fluorescence spectroscopy, reflection spectroscopy, etc. [16,17,18,19,20,21,22]. Of these optical methods, pigment quantification using absorption/transmission spectroscopy with a spectrophotometer stands out as the most cost-effective approach. While rapid methods for determining Fx concentration based on absorbance measurements have been proposed [18,23], their accuracy verification lags behind that of other optical methods that employ flow cytometry with fluorescence [24]. This discrepancy arises from the limited depth of research on the influence of the light scattering effect on the application of the Bouguer–Lambert–Beer (BLB) law in pigment determination. The BLB law serves as the cornerstone of quantification in absorption spectroscopy. However, it has been demonstrated that this law is not applicable to microalgae suspensions due to the light scattering effect [25].

Different models have been proposed as alternatives to the BLB law, such as the modified BLB model (mBLB) [26]. In previous research, a nonlinear model for absorbance was also proposed [25], where different levels of nonlinearity were observed at different wavelengths through the absorbance spectrum of *P. tricornutum*. Extended from the observation, we hypothesized that (1) the level of nonlinearity will also influence the accuracy of the determination of pigment contents when using the BLB model, and (2) the use of the mBLB model is more accurate than the BLB model for pigment content determination.

## 2. Results and Discussion

**Figure 1:**
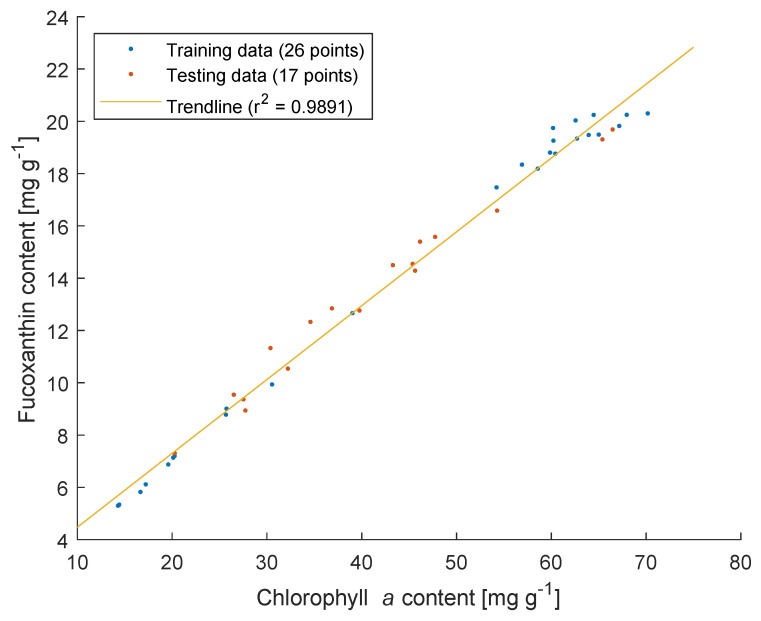
Correlation between chlorophyll *a* and fucoxanthin contents in suspensions of *P. tricornutum* under various light and nitrogen supply conditions.

In the current study, *P. tricornutum* was cultivated under different illumination and nitrogen supply conditions to obtain samples with different Chl *a* and Fx contents. With pigment analysis of the samples with HPLC, a strong positive correlation between Chl *a* and Fx was observed as shown in Figure 1. Similar observations regarding this positive correlation between Chl *a* and Fx have been reported previously for *P. tricornutum* [27] under different indoor and outdoor illumination conditions, as well as for *Tisochrysis lutea* [28] under different nitrogen supplies. This correlation arises from the interdependence of Fx production and FCP formation [29].

### 2.1. Spectral Shift with Pigment Content

**Figure 2:**
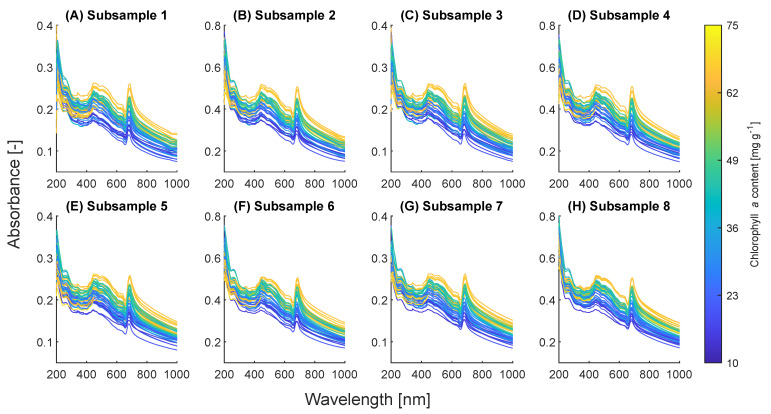
Measured absorbance spectra of all the samples (n=43) in each subsample setting.

As described in Section 3.2, each collected sample was diluted to eight subsamples with different concentrations, and the spectral scans were conducted with different path lengths. The resulting measured net absorbance spectra are depicted in Figure 2, where the spectra are color-coded to represent the corresponding Chl *a* contents. Notably, within the wavelength range of 750 to 1000 nm, where pigment absorption is absent, these spectra primarily reflect pure light scattering. In cases where the cellular characteristics of the samples are reasonably consistent, the spectra within each subsample setting should also exhibit similarity within this scattering range. However, we observed a coefficient of variation ranging from 13.5% to 15.7% for $A_{750}$, indicating substantial dissimilarity among the spectra. Furthermore, a correlation between the spectra and Chl *a* content in this range was observed; specifically, higher Chl *a* content corresponded to higher absorbances, as evident in Figure 2. Light scattering is a complex effect determined by numerous factors, such as cell size, cell weight, morphology, and biochemical compositions [30]. For example, it was reported that the slope of $A_{750}$ to biomass concentration decreases when the cell size increases for *Chlorella vulgaris* [31]. Consequently, we postulated that the spectral shift observed in the current study is attributed to alterations in cellular characteristics induced by different lighting and nitrogen conditions during cultivation. The exact biochemical basis of this spectral shift merits investigation in future research. Importantly, this observed spectral shift poses a challenge to the accuracy of spectrophotometric determination of absolute pigment concentration using Equations (14) or (15), which are also employed in [18]. These equations rely on the consistency of apparent specific absorbances in Equations (4), (5), (7), and (8), and such a spectral shift disrupts this consistency.

### 2.2. Nonlinearity of Absorbances

**Figure 3:**
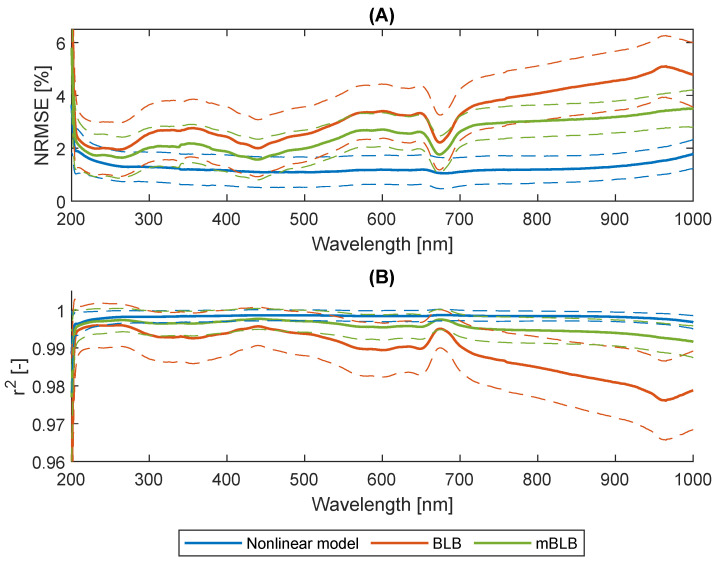
(**A**) NRMSE and (**B**) $r^{2}$ of absorbance prediction with the nonlinear, the Bouguer–Lambert–Beer (BLB), and the modified BLB (mBLB) models. The solid lines are the mean values of all the samples (n=43) and the dashed lines are the standard deviations.

**Figure 4:**
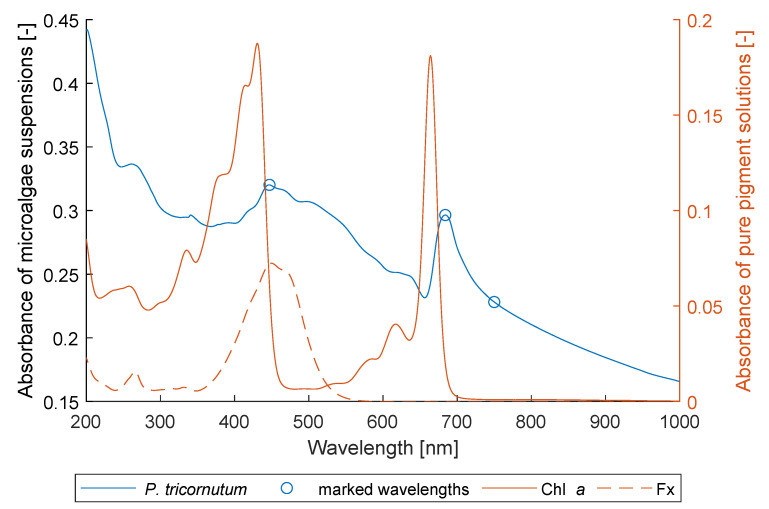
Selection of wavelengths for pigment determination. **Blue**: Average absorbance spectrum of *P. tricornutum* suspensions. **Orange**: Calculated absorbance spectra of pure chlorophyll *a* (Chl *a*) and fucoxanthin (Fx) solutions based on their averaged contents in the samples.

Each collected sample contains eight subsample settings with different concentrations and path lengths. The BLB model (Equation (1)), the mBLB model (Equation (2)), and the nonlinear model (Equation (3)) were used to fit the spectra of eight subsamples in each sample and at each wavelength. The parameters $\epsilon_{\lambda }$, $b_{\lambda }$, $\epsilon_{\lambda }^{\prime }$, $\alpha_{\lambda }$, and $\beta_{\lambda }$ were obtained by solving regression with the models. Our objective was to assess their performance in describing the relationship between absorbance, concentration, and path length. The results, as presented in Figure 3, reveal that the nonlinear model outperforms the others in terms of both the squared Pearson correlation coefficient ($r^{2}$) and the normalized root mean square error (NRMSE). The mBLB model, augmented with an additional intercept term, also exhibits improved descriptive capabilities compared to the BLB model. The current study affirms the suitability of the nonlinear model proposed in [25] for characterizing the nonlinear absorbance patterns observed in light-scattering suspensions. Furthermore, we observe that absorbances characterized by higher pigment absorption tend to yield better accuracies when using both the BLB and mBLB models. This trend is evident in Figure 3. Notably, the accuracy of the BLB model improves significantly around wavelengths of 440 nm and 680 nm, which correspond to the pigment spectra of Chl *a* and Fx in Figure 4.

### 2.3. Wavelengths Selection

Based on the earlier observations, we chose $A_{447}$ and $A_{684}$ for the spectrophotometric determination of pigment contents. These absorbances correspond to the maxima of the wavebands in the averaged spectra shown in Figure 4; hence, they are expected to exhibit the best linearity. Moreover, we selected $A_{750}$ as an additional parameter to provide insights into pure light scattering. As illustrated in Figure 3, $A_{750}$ demonstrates superior accuracy in both $r^{2}$ and NRMSE when compared to $A_{751}$–$A_{1000}$ in both the BLB and mBLB models. This observation indicates that $A_{750}$ exhibits the most favorable linearity. It is noteworthy that our selection of absorbances ($A_{447}$, $A_{684}$, $A_{750}$) is similar to a previous study by Wang et al. [18], where $A_{445}$, $A_{663}$, and $A_{750}$ were employed to determine the absolute concentration of Fx in *P. tricornutum* suspension.

### 2.4. Biomass Concentration Prediction

**Figure 5:**
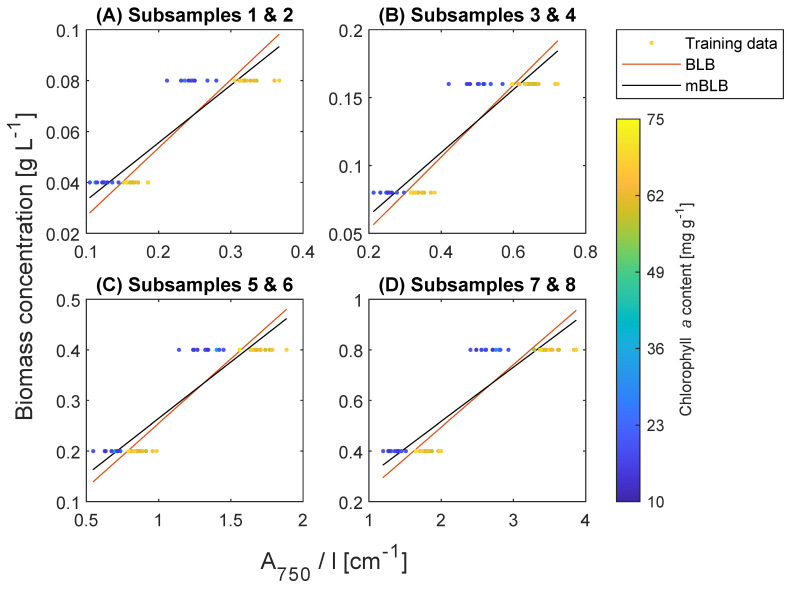
Prediction of biomass concentration with absorbance at 750 nm ($A_{750}$) divided by path length (*l*) using the BLB (Equation (12)) and the modified BLB models (Equation (13)). The data points are colored by their corresponding chlorophyll *a* content.

**Figure 6:**
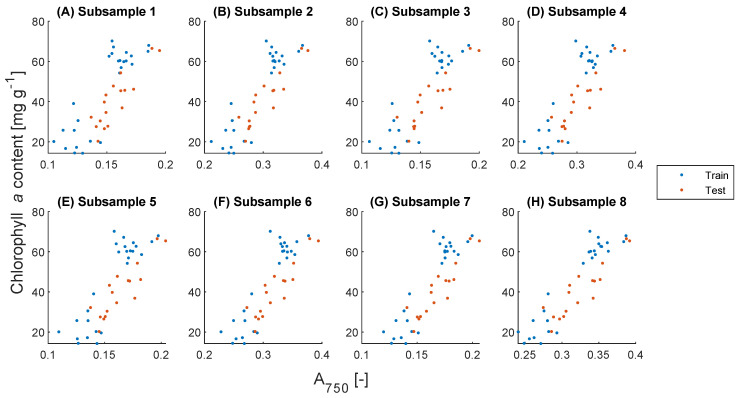
Correlation between chlorophyll *a* and $A_{750}$ in all the subsamples.

Prior to determining pigment content, we obtained the coefficients for biomass concentration prediction in Equations (12) and (13). The results are summarized in Table 1, and notably, there is no substantial distinction between the predictions using the Bouguer–Lambert–Beer (BLB) and the modified BLB (mBLB) models. Figure 5 illustrates the relationship between biomass concentration and $A_{750}/l$, yet the correlation is not entirely clear or satisfactory. In an ideal calibration scenario, the data points for each subsample setting should have converged to the same point since they were all diluted to the same biomass concentration. However, Figure 5 shows a clear trend: higher Chl *a* content corresponds to higher $A_{750}$ for the same concentration. This trend is primarily induced by the spectral shift previously observed in Figure 2. Furthermore, Figure 6 illustrates the correlation between Chl *a* and $A_{750}$ for each subsample. The results in Table 1 and Figure 5 suggest that direct use of $A_{750}$ for biomass concentration estimation is inaccurate when cell optical properties are inconsistent, requiring further corrections. For instance, flow cytometry was used to correct the correlation of biomass concentration and $A_{750}$ for changes in cell size [31].

### 2.5. Pigment Content Prediction

**Figure 7:**
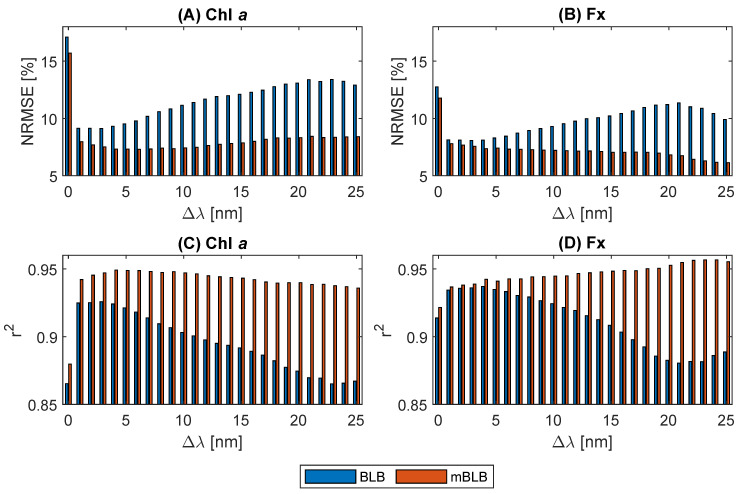
Prediction results of pigment contents with the BLB and the modified BLB model using 3 vs. 5 absorbances as inputs. With 3 absorbances (Δλ=0): $A_{447}$, $A_{684}$, and $A_{750}$. With 5 absorbances (Δλ>0): $A_{447-\Delta \lambda }$, $A_{447+\Delta \lambda }$, $A_{684-\Delta \lambda }$, $A_{684+\Delta \lambda }$, and $A_{750}$. The predictions were performed using the subsamples with the same path lengths (4 cases). For the convenience of comparison, the results of all the cases were combined for the evaluation of $r^{2}$ and NRMSE in the testing dataset (n=136).

Following the acquisition of coefficients for biomass concentration prediction, we proceeded to obtain additional coefficients for predicting Chl *a* and Fx content using Equations (14) and (15) when using three absorbances, as well as Equations (16) and (17) when using five absorbances. To facilitate a straightforward comparison between the use of the BLB model vs. the mBLB model with three vs. five absorbances as inputs, we focused on cases with constant path lengths. The case encompassing all subsamples was excluded from this comparison. More specifically, we conducted separate regression analyses for subsamples 1 and 2, 3 and 4, 5 and 6, and 7 and 8, and subsequently merged all results from the testing datasets (n=136) for the evaluation of $r^{2}$ and NRMSE. The comparative results are depicted in Figure 7, with results using three absorbances as inputs denoted at Δλ=0 and those using five absorbances at Δλ>0.

Figure 7 illustrates a clear advantage in pigment content predictions when employing five absorbances as inputs compared to using only three absorbances, as indicated by the results at Δλ=1 to 10 in comparison to those at Δλ=0. This enhanced performance is attributed to the inherent noise often present in absorbance measurements due to the light scattering effect. The inclusion of additional absorbances serves to mitigate the uncertainty introduced by data noise. Importantly, this improvement in accuracy holds true for both the BLB and mBLB models.

In the case of using five absorbances as inputs, we selected two pairs of wavelengths with equal distances (Δλ) from 447 and 684 nm, along with 750 nm. A closer examination of the results for the BLB model with five absorbances as inputs (Δλ>0) in Figure 7 reveals an interesting trend. Larger values of Δλ lead to decreased prediction accuracy, with this decline persisting until approximately Δλ=20 to 23. This observation suggests that the BLB model performs optimally when the wavelengths of input absorbances are closer to 447 and 684 nm. This finding aligns with the previously noted trend that absorbances with higher pigment absorption yield superior accuracies when using the BLB model, as demonstrated in Figure 3. In contrast, the results for the mBLB model with five absorbances as inputs (Δλ>0) exhibit different characteristics. For Chl *a*, prediction accuracy remains relatively stable from Δλ=1 to 25 in Figure 7a,c. Additionally, the accuracy for Fx increases with larger Δλ in Figure 7b,d. This divergence in behavior between the BLB and mBLB models emphasizes the importance of selecting an appropriate model and absorbances when performing pigment content predictions.

In summary, the mBLB model consistently outperforms the BLB model in all cases for predicting the pigment content of Chl *a* and Fx, as depicted in Figure 7. Despite both models being linear, the mBLB model, with its intercept term, exhibits a greater capacity to account for the influence of light scattering effects. For Chl *a* content prediction, the most accurate results are achieved with the mBLB model using absorbances $A_{441}$, $A_{453}$, $A_{678}$, $A_{690}$, and $A_{750}$ as inputs, whose obtained coefficients are given in Table 2. Similarly, for Fx content prediction, the most accurate results are obtained when using $A_{423}$, $A_{471}$, $A_{660}$, $A_{708}$, and $A_{750}$ as inputs, with the respective coefficients presented in Table 3.

It should be noted that the pigment content prediction results in Figure 7 were analyzed using only the subsamples with constant path lengths for convenience of comparison. The results using all subsamples are also presented in Table 2 and Table 3. Upon a more detailed examination of the data within these tables, it is shown that the results of subsample No. 1 and 2 (l= 1 cm) exhibit slightly superior NRMSE values compared to the other cases (l=0.5, 0.2, and 0.1 cm) for both Chl *a* and Fx content predictions. This phenomenon can be attributed to the fact that higher biomass concentrations lead to a more pronounced light scattering effect when subsamples share the same cl. As shown in Table 4, the odd-numbered subsamples (No. 1, 3, 5, 7) have the same cl, as do the even-numbered subsamples (No. 2, 4, 6, 8). Additionally, Table 2 and Table 3 show that pigment content predictions can be effectively performed by utilizing data from all subsamples collectively. These comprehensive results yield satisfactory outcomes across the various cases. The advantage of employing this approach lies in the fact that the same coefficients obtained from the calibration can be applied to diverse path lengths. This flexibility proves particularly valuable when the intended path length differs from the path lengths employed in the calibration process.

In previous related work by Wang et al. [18], the authors resuspended the cells of *P. tricornutum* in an ethanol solution and employed three absorbances, $A_{445}$, $A_{663}$, and $A_{750}$, to calculate the absolute concentration of Fx. Although they reported $R^{2}$ values exceeding 0.94, other authors documented an $R^{2}$ value of 0.8593 while attempting to verify the same method [24]. Note that the $R^{2}$ here should not be confused with the $r^{2}$, as the former indicates the coefficient of determination and the latter denotes the squared Pearson correlation coefficient. Based on the analysis and observations made in the current study, the inconsistent performance of the method is highly likely to be due to the utilization of absolute pigment concentration as the target, which varies with different cell conditions of the samples. To elaborate, the coefficients obtained during calibration may not remain consistent when a spectral shift, as demonstrated in the current study, occurs. The same inaccuracy was observed when using $A_{750}$ to predict the absolute biomass concentration as previously described in Section 2.4. In this context, the results of pigment content prediction prove to be more reliable than the prediction of absolute pigment concentration. This is because the observed spectral shift exists both in the numerator and denominator in the formulation of Equations (16) and (17), and their disturbances partially cancel each other out. This assertion is supported by a comparison of the NRMSE values for pigment content prediction in Table 2 and Table 3 with the results in Table 1 for the prediction of absolute biomass concentration. Although the coefficients obtained in Table 1 are also used in Equations (16) and (17), the NRMSE for pigment content prediction is less than half of the NRMSE for biomass concentration prediction. The results of the current work not only confirm the aforementioned hypotheses but also establish an experimental framework for enhancing the spectrophotometric determination of pigment contents in microalgae. Please note that, for the pigment prediction methods presented in this study, it is essential to ensure that the selected concentration and path length falls within the calibrated range given in Table 4. For example, when using a 1 cm cuvette, the concentration should be within the range of 0.04 to 0.08 g/L.

## 3. Materials and Methods

**Figure 8:**
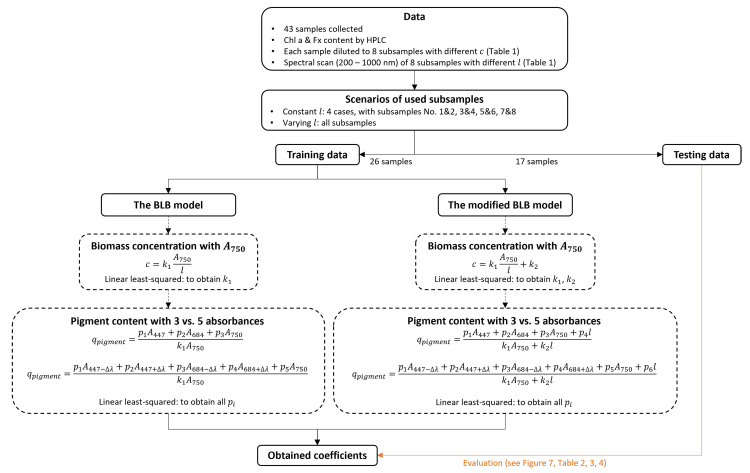
Schematic illustration of the pigment content prediction for chlorophyll *a* and fucoxanthin with the Bouguer–Lambert–Beer (BLB) model and the modified BLB (mBLB) model.

To test our hypotheses, we collected 43 samples of *P. tricornutum* suspensions with varying Chl *a* and Fx contents. Each sample was subsequently diluted into 8 subsamples with different concentrations and the corresponding absorbance spectra were measured with various path lengths to examine the nonlinearity of the absorbances caused by the light scattering effect. The pigment contents of Chl *a* and Fx were measured by HPLC and used to calibrate the models with the measured absorbances. The methods used to predict the pigment content of Chl *a* and Fx in the current study are summarized in Figure 8.

### 3.1. Cultivation of Phaeodactylum tricornutum

The marine microalga *P. tricornutum* SAG 1090-1b was obtained from the Culture Collection of Algae at Göttingen (SAG) and cultivated in four flat-panel airlift (FLA) photobioreactors (PBR) with artificial illumination using LED panels. Two of the FPA-PBRs have a volume of 6 L, and the other two have volumes of 30 L. The FPA-PBRs are equipped with Siemens programmable logic controller (PLC) units to control the light intensity, temperature, pH value, and feeding of substrates. The temperature of the cultivation was maintained at 20 ± 1 ${}^{\circ }C$ and the pH value at 7.3 ± 0.1. The design and the advantages of this type of FPA-PBR system were described in [32,33,34]. A modified Mann and Myers medium was used as the cultivation medium for *P. tricornutum*. This medium was originally designed by Mann and Myers [35] and further modified by Meiser et al. [36]. During the cultivation, solutions of ammonium, phosphate, and trace elements were fed into the culture to provide necessary nutrients and essential elements for the growth of *P. tricornutum*. The details of the cultivation system, culture media, and substrate are described in the work of Derwenskus et al. [37]. To obtain microalgae cells with various pigment contents and cell conditions, *P. tricornutum* was cultivated in a repeated fed-batch process with biomass concentration between 1 and 8 g/L under different combinations of light and nitrogen supplies. The specific light availability ranged from 2 to 8 μmol_photon_ g−1 s−1 and the nitrogen supply varied from N-depletion to N-sufficient. In the case of the N-depletion condition, the ammonium solution was not fed anymore.

### 3.2. Measurement Procedure with UV-Vis Spectrophotometer

The absorbance measurement procedure of *P. tricornutum* in the current work follows the same steps, setups, and measurement devices/equipment as described in [25]. The procedure can be summarized in the following four steps. (1) Centrifugation and resuspension of a collected sample in 0.9% NaCl solution. (2) Determination of biomass concentration by the dry weight method. (3) Dilution of the sample to 8 subsamples with designed concentrations (Table 4). (4) Spectral scans of the subsamples were conducted once between 200 and 1000 nm with a 1 nm interval using a standard spectrophotometer (U-2900, HITACHI, Tokyo, Japan) and quartz cuvettes (100-QS, Hellma, Müllheim, Germany) with different path lengths (Table 4). As for blank measurements, spectral scans (200–1000 nm with 1 nm interval) of 0.9% NaCl solution with all the used cuvettes were carried out at the beginning of the work. These blank measurements were repeated 3 times and the averaged ones were used.

The determination of the biomass concentration of each sample was conducted in duplicate, with the mean value considered as the sample concentration. The coefficient of variation of the analytical duplicates is 1.4%±1.22%. The determination process involved the following steps: (1) Glass-fiber filters (0.2 μm, MN 85/70, Macherey–Nagel GmbH, Düren, Germany) were dried at 105 ${}^{\circ }C$ for a minimum of 24 h and weighed for subsequent use. (2) An amount of 5 mL of the microalgae suspension was filtered through the predried filter on a Büchner funnel and the sample was rinsed twice with 5 mL of tap water to remove the residual medium. (3) The filter containing the microalgae cells was dried using a moisture analyzer (MA35, Sartorius, Göttingen, Germany) at 105 ${}^{\circ }C$ and weighed. (4) Concentration was calculated based on the weight change of the filter.

### 3.3. Determination of Chlorophyll *a* and Fucoxanthin Contents by HPLC

Samples taken during cultivations were washed twice with 0.9% NaCl solution through centrifugations at 4000 rpm for 10 min. The sample pallets were stored at −20 °C and then freeze-dried through lyophilization at −50 °C (Vaco 5, ZIRBUS, Bad Grund, Germany). After lyophilization, 1.5 mL of ethanol containing 250 mg/L of butylated hydroxytoluene (BHT) was added to 20 mg of the lyophilized biomass along with 1 g of Zirkonia glass beads (0.5 mm diameter). The sample was then solubilized in a homogenizer (Precellys^®^ 24, Bertin Technologies, Montigny-le-Bretonneux, France) for three cycles at 5000 rpm for 90 s with 45 s pauses. After centrifugation at 13,000 rpm for 3 min, 1 mL of the extract was collected and replaced with 1 mL of ethanol/BHT. This process was repeated four times to yield a total extract volume of 5 mL. The extract was further clarified by centrifuging at 4000 rpm for 10 min and was then diluted 4 fold in ethanol/BHT for subsequent analysis in HPLC.

Pigment contents were determined by HPLC (Agilent 1200 Infinity, Agilent Technologies, Santa Clara, CA, USA) equipped with a reversed-phase column (Suplex pKb 100, 5 mm, 250 × 4.6 mm, Sigma-Aldrich, Burlington, MA, USA) and a diode array detector (Agilent Technologies). The mobile phase A is constituted of methanol/acetonitrile/2-propanol in a 54/44/2 (*v*/*v*/*v*) ratio. The mobile phase B is constituted of 85% (*v*/*v*) phase A and 15% (*v*/*v*) distilled water. A total of 5 μL of sample volume was injected for analysis and separated by applying a flow rate of 1 μL/min. Chl *a* and Fx were detected at 430, 450, and 460 nm. The applied HPLC gradient with slight modifications was taken from [38] by omitting ethyl acetate. The applied elution procedure started with 40% (*v*/*v*) A and 60% (*v*/*v*) B. After a 10 min linear gradient, 80% (*v*/*v*) A and 20% (*v*/*v*) B were set. The following linear gradient reached 100% (*v*/*v*) A after 20 min. This setting was held constant until reaching 28 min of elution. In the last gradient, the mobile phase was set back to the starting condition within 1 min and held constant for 9 min for conditioning. Quantification was carried out against authentic analytical grade external standards for Chl *a* and Fx.

### 3.4. Data Analysis and Model Fitting

The software Matlab R2022b was used for all the data processing and modeling in the current work. The goodness of fit was evaluated with the squared Pearson correlation coefficient ($r^{2}$) and the normalized root mean square error (NRMSE), which is the RMSE divided by the mean value.

#### 3.4.1. Absorbance Prediction

In the first part of modeling, our primary objective was to further validate the proposed nonlinear model [25] with *P. tricornutum* suspension cultivated under diverse conditions. This nonlinear model had previously demonstrated its capability to accurately characterize nonlinear absorbances across varying concentrations and path lengths. All 43 samples collected in the current work were used in this part of the modeling. The model fitting was conducted for each sample with the spectra of 8 diluted subsamples (see Table 4). The regression was conducted with the Matlab function “fit” using the following three models (Equations (1)–(3)).

The Bouguer–Lambert–Beer (BLB) model:

(1) $$A_{\lambda }=A_{\lambda ,suspension}-A_{\lambda ,blank}=\epsilon_{\lambda }\;\cdot \;c\;\cdot \;l$$

Here, $A_{\lambda }$ [-] is the net absorbance at λ nm, $A_{\lambda ,suspension}$ (-) is the measured absorbance of *P. tricornutum* suspension, $A_{\lambda ,blank}$ (-) is the measured absorbance of 0.9% NaCl solution, $\epsilon_{\lambda }$ (L g^−1^ cm^−1^) is the apparent specific absorbance, *c* (g L^−1^) is the biomass concentration, and *l* (cm) is the path length.

The modified BLB (mBLB) model:

(2) $$A_{\lambda }=\epsilon_{\lambda }\;\cdot \;c\;\cdot \;l+b_{\lambda }\;\cdot \;l$$

Here, $A_{\lambda }$, $\epsilon_{\lambda }$, *c*, and *l* are the same as above. $b_{\lambda }$ is an intercept term (cm^−1^).

The nonlinear model:

(3) $$A_{\lambda }=\epsilon_{\lambda }^{\prime }\;\cdot \;c^{\alpha_{\lambda }}\;\cdot \;l^{\beta_{\lambda }}$$

Here, $A_{\lambda }$, *c*, and *l* are the same as above. $\alpha_{\lambda }$ and $\beta_{\lambda }$ are correction constants (-) and $\epsilon_{\lambda }^{\prime }$ is the effective specific absorbance, whose unit depends on $\alpha_{\lambda }$ and $\beta_{\lambda }$.

#### 3.4.2. Pigment Content Prediction

In the second and most important part of the modeling, the objective was to analyze the use of the BLB and modified BLB models in the determination of Chl *a* and Fx content in *P. tricornutum* suspensions. The subsamples of 26 samples were used as a training dataset to calibrate the parameters and the subsamples of the other 17 samples were used as a testing dataset to evaluate the performance of the predictions. Initially, 26 samples were collected for pigment prediction, yielding satisfactory preliminary results. Subsequently, 17 additional samples were taken. After confirming that their pigment concentrations were within the range of the original 26 samples (Figure 1), these 17 samples were selected as testing data and the original 26 samples as training data. Unlike most other research where only 1 cm of path length was used for the measurements, different path lengths were systematically used in the current work. Therefore, the mathematical description of the pigment determination was formulated in a more general way that takes into account the change in path length. Meanwhile, we ran the regressions with the training data at the same path length (4 cases: subsample 1 and 2, 3 and 4, 5 and 6, and 7 and 8) and at different path lengths (all subsamples). The models were then evaluated with the testing dataset using the same subsamples as in the regressions. The classical linear least-squares method was used to determine the coefficients of the models.

To select the wavelengths for spectrophotometric determination of Chl *a* and Fx content, we first averaged all obtained spectra of all samples (n=43) with all subsample settings (n=8) as shown in Figure 4. A broad waveband with maximum absorption at 447 nm was observed, corresponding to the mixed spectrum of pure Chl *a* and Fx solutions. Another narrow waveband with maximum absorption at 684 nm was observed at 684 nm, which corresponds to the narrow waveband of the spectrum of the pure Chl *a* solution.

Therefore, we chose the absorbance values at 447, 684, and 750 nm as inputs for pigment content determination. The absorbance values at 447 and 684 nm contain the spectral information of Chl *a* and Fx. The absorbance at 750 nm contains the background information of the cells without Chl *a* and Fx. Applying the principles of multicomponent spectrophotometric analysis [39], the following linear equations are formulated using the BLB and the modified BLB models.

Linear equations using the BLB model:

(4) $$A_{447}=\epsilon_{11}c_{1}l+\epsilon_{12}c_{2}l+\epsilon_{13}c_{3}l$$

(5) $$A_{684}=\epsilon_{21}c_{1}l+\epsilon_{22}c_{2}l+\epsilon_{23}c_{3}l$$

(6) $$A_{750}=\epsilon_{3}cl$$

Linear equations using the mBLB model:

(7) $$A_{447}=\epsilon_{11}c_{1}l+\epsilon_{12}c_{2}l+\epsilon_{13}c_{3}l+b_{447}l$$

(8) $$A_{684}=\epsilon_{21}c_{1}l+\epsilon_{22}c_{2}l+\epsilon_{23}c_{3}l+b_{684}l$$

(9) $$A_{750}=\epsilon_{3}cl+b_{750}l$$

Here, $\epsilon_{ij}$ (L g^−1^ cm^−1^) is the apparent specific absorbance at the *i*th wavelength for the *j*th component. $c_{j}$ (g L^−1^) is the concentration of the *j*th component, where $c_{1}$ is the Chl *a* concentration, $c_{2}$ the Fx concentration, and $c_{3}$ the concentration of the other components in biomass except Chl *a* and Fx. The parameter *c* is the biomass concentration and follows the equation $c=c_{1}+c_{2}+c_{3}$. The parameter $\epsilon_{3}$ is the apparent specific absorbance at 750 nm corresponding to *c*. The parameters $A_{\lambda }$, $b_{\lambda }$, and *l* are the same as above. Note that Equations (6) and (9) were formulated with *c* for simplicity, as the absorbances of pigments at 750 nm are almost nonexistent.

By solving Equations (4)–(6), the Chl *a* or Fx concentration ($c_{1}$ or $c_{2}$) can be formulated and reorganized as $c_{pigment}$ (mg L^−1^) in Equation (10) with coefficients $p_{1}$ to $p_{3}$ (mg cm L^−1^) using the BLB model.

(10) $$c_{pigment}=\frac{p_{1}A_{447}+p_{2}A_{684}+p_{3}A_{750}}{l}$$

By solving Equations (7)–(9), the Chl *a* or Fx concentration ($c_{1}$ or $c_{2}$) can be formulated and reorganized as $c_{pigment}$ (mg L^−1^) in Equation (11) with coefficients $p_{1}$ to $p_{3}$ (mg cm L^−1^) and $p_{4}$ (mg L^−1^) using the mBLB model.

(11) $$c_{pigment}=\frac{p_{1}A_{447}+p_{2}A_{684}+p_{3}A_{750}}{l}+p_{4}$$

By reorganizing Equations (6) and (9), the biomass concentration, *c* (g L^−1^), can be expressed as Equations (12) and (13), respectively. Here, $k_{1}$ (g cm L^−1^) and $k_{2}$ (g L^−1^) are calibration coefficients.

Biomass concentration prediction with $A_{750}$ using the BLB model:

(12) $$c=k_{1}\frac{A_{750}}{l}$$

Biomass concentration prediction with $A_{750}$ using the mBLB model:

(13) $$c=k_{1}\frac{A_{750}}{l}+k_{2}$$

Finally, the pigment content, $q_{pigment}$ (mg g^−1^), for Chl *a* or Fx is formulated in Equation (14) by combining Equations (10) and (12) using the BLB model. Equations (11) and (13) are combined for the case using the mBLB model as described in Equation (15). To obtain the involved coefficients, the $k_{1}$ in Equation (12) and the $k_{1}$ and $k_{2}$ in Equation (13) were first determined by the classical linear least-squares method. Afterwards, the $p_{1}$ to $p_{3}$ of Equation (14) and the $p_{1}$ to $p_{4}$ of Equation (15) were obtained by the classical linear least-squares method.

Pigment content prediction with 3 absorbances using the BLB model:

(14) $$q_{pigment}=\frac{c_{pigment}}{c}=\frac{p_{1}A_{447}+p_{2}A_{684}+p_{3}A_{750}}{k_{1}A_{750}}$$

Pigment content prediction with 3 absorbances using the mBLB model:

(15) $$q_{pigment}=\frac{p_{1}A_{447}+p_{2}A_{684}+p_{3}A_{750}+p_{4}l}{k_{1}A_{750}+k_{2}l}$$

Because the spectral measurements of microalgae suspensions are usually noisy due to the light scattering effect, we expected that the pigment content prediction would be more accurate by including additional absorbances. To achieve this, we used 5 absorbances as inputs in the pigment content prediction, as formulated in Equations (16) and (17) with coefficients $p_{1}$ to $p_{5}$ (mg cm L ^−1^) and $p_{6}$ (mg L ^−1^). Two pairs of absorbances at the same distance from the maximum of wavebands ($A_{447}$ and $A_{684}$) with a span of Δλnm along with $A_{750}$ were included as inputs. Here, we first determined the $k_{1}$ in Equation (12) and the $k_{1}$ and $k_{2}$ in Equation (13) by the classical linear least-squares method and then determined the $p_{1}$ to $p_{5}$ of Equation (16) and the $p_{1}$ to $p_{6}$ of Equation (17).

Pigment content prediction with 5 absorbances using the BLB model:

(16) $$q_{pigment}=\frac{p_{1}A_{447-\Delta \lambda }+p_{2}A_{447+\Delta \lambda }+p_{3}A_{684-\Delta \lambda }+p_{4}A_{684+\Delta \lambda }+p_{5}A_{750}}{k_{1}A_{750}}$$

Pigment content prediction with 5 absorbances using the mBLB model:

(17) $$q_{pigment}=\frac{p_{1}A_{447-\Delta \lambda }+p_{2}A_{447+\Delta \lambda }+p_{3}A_{684-\Delta \lambda }+p_{4}A_{684+\Delta \lambda }+p_{5}A_{750}+p_{6}l}{k_{1}A_{750}+k_{2}l}$$

## 4. Conclusions

The findings of the current study validate our hypotheses: (1) The accuracy of predicting pigment content (Chl *a* and Fx) using the BLB model decreases with higher nonlinearity of the absorbances, and (2) the modified BLB model outperforms the BLB model in predicting pigment content. Furthermore, our results demonstrate that using five absorbances as inputs yields superior predictions compared to using three absorbances. It is also suggested that predicting pigment content is a more suitable approach than predicting absolute pigment concentration. The method developed in the current study can be used for rapid determination of Chl *a* and Fx content with *P. tricornutum* suspension. Moreover, it has the potential to be extended for pigment prediction in various microalgae species and can serve as the basis for online monitoring of pigment content. Note that the nonlinear model in Equation (3) is not yet involved in the pigment determination, as the assumption of linear superposition of the absorbances is too simplistic for the nonlinear model, which has been motivated by nonlinear light scattering effects. Derivation of a suitable nonlinear superposition model law is the subject of future work.

## Figures and Tables

**Table 1:** Obtained coefficients of biomass concentration prediction with the Bouguer–Lambert–Beer (BLB), and the modified BLB (mBLB) model, as shown in Equations (12) and (13). The $r^{2}$ and the NRMSE are for the testing dataset.

			BLB			mBLB			
**Subsample**	$n_{\mathrm{Train}}$	$n_{\mathrm{Test}}$	$k_{1}$	$r^{2}$	**NRMSE**	$k_{1}$	$k_{2}$	$r^{2}$	**NRMSE**
No. 1 and 2	52	34	0.2678	0.8894	0.1181	0.2262	0.0103	0.8894	0.1200
No. 3 and 4	52	34	0.2655	0.8828	0.1222	0.2319	0.0084	0.8828	0.1204
No. 5 and 6	52	34	0.2547	0.8963	0.1168	0.2228	0.0083	0.8963	0.1148
No. 7 and 8	52	34	0.2472	0.8978	0.1108	0.2139	0.0089	0.8978	0.1125
All	208	136	0.2492	0.9761	0.1431	0.2156	0.0090	0.9748	0.1442

**Table 2:** Obtained coefficients of chlorophyll *a* content prediction with the mBLB model using absorbances $A_{441}$, $A_{453}$, $A_{678}$, $A_{690}$, and $A_{750}$ as inputs (see Equation (17), Δλ=6). The $r^{2}$ and the NRMSE are for the testing dataset.

Subsample	$n_{\mathrm{Train}}$	$n_{\mathrm{Test}}$	$p_{1}$	$p_{2}$	$p_{3}$	$p_{4}$	$p_{5}$	$p_{6}$	$r^{2}$	NRMSE
No. 1 and 2	52	34	−114.23	104.91	−44.44	94.93	−39.58	0.16	0.9629	0.0630
No. 3 and 4	52	34	−134.90	125.88	−37.10	86.37	−38.71	0.30	0.9532	0.0715
No. 5 and 6	52	34	−73.16	63.06	−69.52	115.61	−33.52	0.61	0.9415	0.0769
No. 7 and 8	52	34	−71.53	62.81	−79.37	118.50	−26.85	1.04	0.9435	0.0796
All	208	136	−77.27	69.34	−68.79	111.91	−32.48	0.14	0.9467	0.0749

**Table 3:** Obtained coefficients of fucoxanthin content prediction with the mBLB model using absorbances $A_{423}$, $A_{471}$, $A_{660}$, $A_{708}$, and $A_{750}$ as inputs (see Equation (17), Δλ=24). The $r^{2}$ and the NRMSE are for the testing dataset.

Subsample	$n_{\mathrm{Train}}$	$n_{\mathrm{Test}}$	$p_{1}$	$p_{2}$	$p_{3}$	$p_{4}$	$p_{5}$	$p_{6}$	$r^{2}$	NRMSE
No. 1 and 2	52	34	−15.07	15.86	−19.15	10.39	9.90	0.06	0.9597	0.0581
No. 3 and 4	52	34	−11.33	12.63	−21.52	12.12	9.80	0.06	0.9599	0.0600
No. 5 and 6	52	34	−10.89	11.96	−20.61	11.50	9.66	0.09	0.9552	0.0660
No. 7 and 8	52	34	−6.21	9.96	−31.23	4.93	24.42	0.17	0.9565	0.0628
All	208	136	−9.70	10.61	−20.13	12.56	8.24	0.07	0.9515	0.0651

**Table 4:** Setups of biomass concentration (*c*) and path length (*l*) for subsamples.

Subsample	No. 1	No. 2	No. 3	No. 4	No. 5	No. 6	No. 7	No. 8
*c* (g/L^−1^)	0.04	0.08	0.08	0.16	0.2	0.4	0.4	0.8
*l* (cm)	1	1	0.5	0.5	0.2	0.2	0.1	0.1

## Data Availability

The data presented in the current study are available on request from the corresponding author.

## Abbreviations

The following abbreviations are used in this manuscript:

Chl *a*	Chlorophyll *a*
Fx	Fucoxanthin
FCP	Fucoxanthin chlorophyll *a*/*c* binding protein
HPLC	High-performance liquid chromatography
BHT	Butylated hydroxytoluene
BLB	The Bouguer–Lambert–Beer law
mBLB	The modified Bouguer–Lambert–Beer model
SAG	The Culture Collection of Algae at Göttingen
FPA	Flat-panel airlift
PBR	Photobioreactor
PLC	Programmable logic controller
$r^{2}$	The squared Pearson correlation coefficient
$R^{2}$	The coefficient of determination
NRMSE	The normalized root mean square error

## References

[1] Li Y., Chen M., Li Y., Chen M. (2015). Novel chlorophylls and new directions in photosynthesis research. Funct. Plant Biol..

[2] Lehmuskero A., Skogen Chauton M., Boström T. (2018). Light and photosynthetic microalgae: A review of cellular- and molecular-scale optical processes. Prog. Oceanogr..

[3] Kuczynska P., Jemiola-Rzeminska M., Nowicka B., Jakubowska A., Strzalka W., Burda K., Strzalka K. (2020). The xanthophyll cycle in diatom Phaeodactylum tricornutum in response to light stress. Plant Physiol. Biochem..

[4] Wang W., Yu L.J., Xu C., Tomizaki T., Zhao S., Umena Y., Chen X., Qin X., Xin Y., Suga M. (2019). Structural basis for blue-green light harvesting and energy dissipation in diatoms. Science.

[5] da Silva Ferreira V., Sant’Anna C. (2017). Impact of culture conditions on the chlorophyll content of microalgae for biotechnological applications. World J. Microbiol. Biotechnol..

[6] Rocha D.H.A., Pinto D.C.G.A., Silva A.M.S. (2022). Macroalgae Specialized Metabolites: Evidence for Their Anti-Inflammatory Health Benefits. Mar. Drugs.

[7] Winarto J., Song D.G., Pan C.H. (2023). The Role of Fucoxanthin in Non-Alcoholic Fatty Liver Disease. Int. J. Mol. Sci..

[8] Sayuti N.H., Muhammad Nawawi K.N., Goon J.A., Mokhtar N.M., Makpol S., Tan J.K. (2023). A Review of the Effects of Fucoxanthin on NAFLD. Nutrients.

[9] Kim S.M., Jung Y.J., Kwon O.N., Cha K.H., Um B.H., Chung D., Pan C.H. (2012). A Potential Commercial Source of Fucoxanthin Extracted from the Microalga Phaeodactylum tricornutum. Appl. Biochem. Biotechnol..

[10] Bigagli E., D’Ambrosio M., Cinci L., Niccolai A., Biondi N., Rodolfi L., Dos Santos Nascimiento L.B., Tredici M.R., Luceri C. (2021). A Comparative In Vitro Evaluation of the Anti-Inflammatory Effects of a Tisochrysis lutea Extract and Fucoxanthin. Mar. Drugs.

[11] Lee A.H., Shin H.Y., Park J.H., Koo S.Y., Kim S.M., Yang S.H. (2021). Fucoxanthin from microalgae Phaeodactylum tricornutum inhibits pro-inflammatory cytokines by regulating both NF-κB and NLRP3 inflammasome activation. Sci. Rep..

[12] Pajot A., Hao Huynh G., Picot L., Marchal L., Nicolau E. (2022). Fucoxanthin from Algae to Human, an Extraordinary Bioresource: Insights and Advances in up and Downstream Processes. Mar. Drugs.

[13] Derwenskus F., Weickert S., Lewandowski I., Schmid-Staiger U., Hirth T. (2020). Economic evaluation of up- and downstream scenarios for the co-production of fucoxanthin and eicosapentaenoic acid with *P. tricornutum* using flat-panel airlift photobioreactors with artificial light. Algal Res..

[14] Lichtenthaler H.K. (1987). [34] Chlorophylls and carotenoids: Pigments of photosynthetic biomembranes. Methods in Enzymology.

[15] Gille A., Trautmann A., Posten C., Briviba K. (2016). Bioaccessibility of carotenoids from Chlorella vulgaris and Chlamydomonas reinhardtii. Int. J. Food Sci. Nutr..

[16] Eriksen N.T., Iversen J.J.L. (1995). On-line determination of pigment composition and biomass in cultures of microalgae. Biotechnol. Technol..

[17] Shao Y., Pan J., Zhang C., Jiang L., He Y. (2015). Detection in situ of carotenoid in microalgae by transmission spectroscopy. Comput. Electron. Agric..

[18] Wang L.J., Fan Y., Parsons R., Hu G.R., Zhang P.Y., Li F.L. (2018). A Rapid Method for the Determination of Fucoxanthin in Diatom. Mar. Drugs.

[19] Pilát Z., Bernatová S., Ježek J., Šerý M., Samek O., Zemánek P., Nedbal L., Trtílek M. (2012). Raman microspectroscopy of algal lipid bodies: β-carotene quantification. J. Appl. Phycol..

[20] Gao F., Sá M., Teles (Cabanelas, ITD) I., Wijffels R.H., Barbosa M.J. (2021). Production and monitoring of biomass and fucoxanthin with brown microalgae under outdoor conditions. Biotechnol. Bioeng..

[21] Lian L., Hu X., Huang Z., Hu L., Xu L. (2021). Pigment analysis based on a line-scanning fluorescence hyperspectral imaging microscope combined with multivariate curve resolution. PLoS ONE.

[22] Vinh T.Q., Trung T.N., Balasus J., Sharma S., Hegemann T., Greulich S., Khanh T.Q., Kaldenhoff R. (2021). Light reflection spectra as a tool for direct and real-time determination of biomass and pigments in the microalgae Microchloropsis salina. Light. Res. Technol..

[23] Thrane J.E., Kyle M., Striebel M., Haande S., Grung M., Rohrlack T., Andersen T. (2015). Spectrophotometric Analysis of Pigments: A Critical Assessment of a High-Throughput Method for Analysis of Algal Pigment Mixtures by Spectral Deconvolution. PLoS ONE.

[24] Macdonald Miller S., Abbriano R.M., Segecova A., Herdean A., Ralph P.J., Pernice M. (2021). Comparative Study Highlights the Potential of Spectral Deconvolution for Fucoxanthin Screening in Live Phaeodactylum tricornutum Cultures. Mar. Drugs.

[25] Yeh Y.C., Haasdonk B., Schmid-Staiger U., Stier M., Tovar G.E.M. (2023). A novel model extended from the Bouguer-Lambert-Beer law can describe the non-linear absorbance of potassium dichromate solutions and microalgae suspensions. Front. Bioeng. Biotechnol..

[26] Kocsis L., Herman P., Eke A. (2006). The modified Beer–Lambert law revisited. Phys. Med. Biol..

[27] Butler T.O., Padmaperuma G., Lizzul A.M., McDonald J., Vaidyanathan S. (2022). Towards a Phaeodactylum tricornutum biorefinery in an outdoor UK environment. Bioresour. Technol..

[28] Gao F., Teles I., Ferrer-Ledo N., Wijffels R.H., Barbosa M.J. (2020). Production and high throughput quantification of fucoxanthin and lipids in Tisochrysis lutea using single-cell fluorescence. Bioresour. Technol..

[29] Truong T.Q., Park Y.J., Koo S.Y., Choi J.H., Enkhbayar A., Song D.G., Kim S.M. (2023). Interdependence of fucoxanthin biosynthesis and fucoxanthin-chlorophyll a/c binding proteins in Phaeodactylum tricornutum under different light intensities. J. Appl. Phycol..

[30] Morel A., Bricaud A. (1981). Theoretical results concerning light absorption in a discrete medium, and application to specific absorption of phytoplankton. Deep Sea Res. Part A Oceanogr. Res. Pap..

[31] Chioccioli M., Hankamer B., Ross I.L. (2014). Flow Cytometry Pulse Width Data Enables Rapid and Sensitive Estimation of Biomass Dry Weight in the Microalgae Chlamydomonas reinhardtii and Chlorella vulgaris. PLoS ONE.

[32] Schmid-Staiger U., Preisner R., Trösch W., Marek P. (2009). Kultivierung von Mikroalgen im Photobioreaktor zur stofflichen und energetischen Nutzung. Chem. Ing. Tech..

[33] Bergmann P., Ripplinger P., Beyer L., Trösch W. (2013). Disposable Flat Panel Airlift Photobioreactors. Chem. Ing. Tech..

[34] Bergmann P., Trösch W. (2016). Repeated fed-batch cultivation of Thermosynechococcus elongatus BP-1 in flat-panel airlift photobioreactors with static mixers for improved light utilization: Influence of nitrate, carbon supply and photobioreactor design. Algal Res..

[35] Mann J.E., Myers J. (1968). On Pigments, Growth, and Photosynthesis of Phaeodactylum Tricornutum12. J. Phycol..

[36] Meiser A., Schmid-Staiger U., Trösch W. (2004). Optimization of eicosapentaenoic acid production by Phaeodactylum tricornutumin the flat panel airlift (FPA) reactor. J. Appl. Phycol..

[37] Derwenskus F., Schäfer B., Müller J., Frick K., Gille A., Briviba K., Schmid-Staiger U., Hirth T. (2020). Coproduction of EPA and Fucoxanthin with P. tricornutum—A Promising Approach for Up- and Downstream Processing. Chem. Ing. Tech..

[38] Kraay G.W., Zapata M., Veldhuis M.J.W. (1992). Separation of Chlorophylls C1c2, and C3 of Marine Phytoplankton by Reversed-Phase-C18-High-Performance Liquid Chromatography1. J. Phycol..

[39] Kramer R. (1998). Chemometric Techniques for Quantitative Analysis.

